# TOX regulates T lymphocytes differentiation and its function in tumor

**DOI:** 10.3389/fimmu.2023.990419

**Published:** 2023-03-09

**Authors:** Haiyue Niu, Huaquan Wang

**Affiliations:** Department of Hematology, General Hospital, Tianjin Medical University, Tianjin, China

**Keywords:** TOX, CD4, CD8, T-cell exhaustion, tumor

## Abstract

Thymocyte selection-associated high mobility group box protein (TOX) is expressed differently at all T lymphocytes development stages. Owing to more advanced scientific and technological means, including single-cell sequencing technology, heterogeneity of T lymphocytes and TOX has gradually been revealed. Further exploration of such heterogeneity will help us comprehend the developmental stage and functional characteristics of T lymphocytes in greater detail. Emerging evidence supports its regulation not only in exhausting, but also in activating T lymphocytes, thereby verifying TOX heterogeneity. TOX can be used not only as a latent intervention target for tumor diseases and chronic infections, and a therapeutic strategy for autoimmune diseases, but also as a critical factor predicting the drug response and overall survival of patients with malignant tumors.

## 1 Introduction

*TOX* encodes a nuclear HMG protein known as thymocyte selective associated high mobility group box protein (1). TOX was initially identified as a vital regulator that either directly or indirectly regulates thymocyte selection (2).

*TOX* is significantly enriched in humans and mice, with its mRNA being the most highly expressed in the thymus, followed by liver and brain (2). It plays an important role in the formation of CD4^+^ T lymphocytes (3, 4), innate lymphoid cells (ILCs) (5), natural killer (NK) cells (6), and follicular helper T cells (Tfh) (7). While on the one hand, TOX leads to T lymphocytes differentiation, development, effector function, long-term survival, and maintenance, it promotes immune exhaustion and tumorigenesis on the other hand. This review evaluates the effect of TOX on the trajectory of hematopoietic stem cells (HSCs) that develop into mature T lymphocytes and the significance of TOX as a promising target for chronic infection or tumor therapy.

## 2 TOX expression during thymocyte development

### 2.1 Upregulation of TOX is the result of β-selection

T lymphocytes develop in a highly structured sequence under the control of a series of complex and precise regulatory mechanisms. Mouse fetal livers contain abundant Tox^+^ cells, suggesting that HSCs express TOX (8). HSCs gradually differentiate into lymphoid progenitor cells, and move into the thymus, where they finally differentiate into early thymic progenitor (ETP) cells. The number of ETPs differentiating into double negative (DN) thymocytes is not reduced in TOX^-/-^mice. Overall, thymus expresses high levels of TOX, whereas most immature thymocytes do not express much TOX (4). Based on the expression of CD44 and CD25, mouse DN cells can be divided into four stages-D1 to D4-with the expression of CD25 incipiently increasing and then decreasing, whereas that of CD44 decreases gradually. TOX is highly expressed in the early stages (DN1 and DN2), whereas, only a small number of thymocytes in the DN3 and DN4 stages express TOX. β-selection, T-cell receptor (TCR) β-chain gene rearrangement, and pre-TCR expression, occur at the DN3 stage. TOX^+^ T thymocytes may undergo β-selection (4). Importantly, TOX can initiate adjuvant changes associated with β-selection. It has been previously reported that TOX is extensively enriched during the third DN stage owing to β-selection, followed by a downward trend prior to the double positive (DP) phase (2). *TOX*-TG mice are established in the context of RAG deficiency (RAGO). The growth of RAGO thymocytes is blocked in the DN3 stage, and thymocyte density is significantly decreased as β-selection cannot proceed smoothly. Although the thymocyte density in TOX-TG/RAGO mice was similar to that in RAGO mice, all DN3 (CD25^+^CD44^-^) cells express TOX due to the expression of the TOX co-receptor, suggesting that some of the cells express TOX owing to β-selectivity. In the late stage of DN4, TOX expression is downregulated. Mice with recombinant activator gene/recombination-activating gene (*RAG*) deficiency thymocytes—that cannot encode the TCRβ chain and thus arrest cells in the double-negative phase—do not express TOX. Further, TOX was reported to be upregulated post CD3 ε treatment, further indicating that TOX upregulation is the result of β-selection (4).

### 2.2 TOX induces positive selection

After β selection, positive selection plays a role in the subsequent development phase, which requires TCR chain rearrangement and TCR-mediated self-major histocompatibility complex (MHC) recognition to induce DP thymocyte survival and further differentiation and maturation. The expression of TOX was strictly regulated with an increase in the beginning and reduction in the counts of later mature CD4 single positive (SP) and CD8SP thymocytes during positive selection. Positive selection requires mitogen-activated protein kinase (MAPK) and calcineurin (Cn)-mediated pathway activation, where Cn-mediated signaling pathway upregulates TOX during the initial phase of positive selection (9). DP thymocytes in the normal human thymic cortex demonstrate strong TOX staining (8). When DP thymocytes develop into SP thymocytes, TOX is transiently upregulated *via* Cn-mediated TCR signal transduction. *TOX* ^-/-^ thymocytes can successfully complete positive selection; however, the subsequent formation of CD4^+^C8^lo^ and CD4SP thymocytes is hindered (4). CD69—expressed on thymocyte surface in mice—is a marker of ongoing positive selection, during which CD69^+^DP thymocytes express significantly higher TOX than CD69^-^ thymocytes. Normal positive selection still exists in *TOX* ^-/-^ mice, which indicates that the TCR phenotype in the mutant thymocytes is unchanged, and TOX does not affect TCR signal transduction, but CD4SP lineage fails to differentiate such that the counts of CD4^+^CD8^lo^ and CD4SP thymocytes decrease in the fourth stage. Even in a normal bone marrow microenvironment, *TOX* ^-/-^mice are unable to develop abnormal CD4SP thymocytes. In the *Tox* ^-/-^double dull (DD) thymocyte population, the decreased apoptotic cell counts and Bim expression may be attributed to the dilution of post-positive selection DD thymocytes and/or lack of thymocytes in CD4, which are usually negatively selected.

### 2.3 TOX is essential for CD4 lineage commitment

TOX is essential for commitment to CD4 lineage. *Tox* ^-/-^ mice demonstrate a dramatic decrease in the number of lymphocytes, predominantly CD4^+^ thymocytes. ThPok, which is encoded by the zinc finger and BTB domain containing 7B(*Zbtb7b*) gene, leads to CD4SP lymphocyte formation. Sustained ThPok expression is the key to maintaining *CD8* silencing in CD4^+^ thymocytes. ThPok deletion or miscoding leads to CD8SP lineage development, whereas ectopic expression of ThPok leads to the generation of CD4SP lineage (10). Inhibition of CD8 by ThPok does not depend on TOX. Compared with wild-type (WT) mouse, ThPok mRNA expression in CD4^+^ thymocytes in TOX^-/-^ mice is markedly reduced. GATA binding protein 3(*GATA3*) expressed higher in CD4SP thymocytes, alternatively, is more like a regulator targeting ThPok. In mouse with *TOX* is knockout, although positive selection can be initiated and GATA3 expression is upregulated, the expression of ThPOK cannot be induced (3).

Activated Naive CD4^+^T lymphocytes differentiate into Tfh cells and involved in humoral immune defense. All programmed cell death protein 1(PD-1) ^+^Tfh cells in normal individuals strongly express TOX (8). Further, heterotopic expression of TOX in T lymphocytes increases the number of Tfh cells, while TOX downregulation weakens the ability of Tfh cells to respond to antigens. In mouse and human Tfh cells, elevated TOX expression is driven by *B cell leukemia/lymphoma 6 (BCL-6)*. Correspondingly, TOX promotes the expression of a variety of critical molecules (e.g., T cell factor 1(*TCF-1*), lymphoid enhancer binding factor 1(*LEF1*), *PD-1*) to control the growth, differentiation, and biological effects of Tfh cells (11).

### 2.4 Heterogeneous function in CD8 lineage

#### 2.4.1 Forced induction of TOX is sufficient to induce CD8SP lymphocyte activation

TOX in mature CD8^+^T lymphocytes is majorly regulated by the Cn-mediated signaling pathway (9). Although the splenic structure was normal in *TOX* ^-/-^ mice, the counts of CD8^+^T lymphocytes demonstrated a slight reduction, indicating that TOX was not necessary for CD8 lineage maturation. In CD8^-^TOX^-^ cells, forced expression of TOX was sufficient to induce CD8TOXαβ upregulation. The counts of TCR^+^CD8SP cells in Tg-TOX (CD8^-^TOX^-^ cells whose counts were strongly induced in response to TOX expression) were increased, whereas those of CD4SP thymocytes decreased, which were not regulated by MHC class I or II molecules, suggesting that TOX was sufficient to initiate the induction of CD8 lineage maturation (2). The differentiation of CD8SP lymphocytes requires CD4 silencing, and the positive selection mediated by TOX is associated with the upregulation of RUNX family transcription factor 3 (*Runx3*) or the alteration of chromatin structure. Additionally, transformation of DP cells to CD8SP cells requires CD8 gene activation, which requires CD8 demethylation. Partial demethylation of CD8 was observed in TOX-Tg/RAGo thymocytes expressing CD8 (9). In summary, although TOX is not a necessary factor for cell maturation, TOX expression can support CD8 lineage development.

#### 2.4.2 TOX exhausts CD8^+^ T lymphocytes

Upon antigen presentation, inactivated CD8SP lymphocytes (naïve T) are stimulated and gradually activated to differentiate into cytotoxic T lymphocytes (CTLs). Once the antigen is cleared, CTLs are transformed into memory T lymphocytes. If the antigen continues to persist (e.g., as in case of chronic infection or tumor), continuously activated CD8^+^T lymphocytes progress toward an exhausted phenotype—charactarized by transcriptional and epigenetic changes, increased inhibitory receptors (IRs) expression, decreased functional molecules (e.g., perforin, granzyme, interferon-γ (INF-γ), and tumor necrosis factor-β (TNF-β)), and decreased cell proliferation capacity (interleukin-2 (IL-2) downregulation)—thus significantly reducing the killing effect on pathogens or tumors (12).

**Figure 1:**
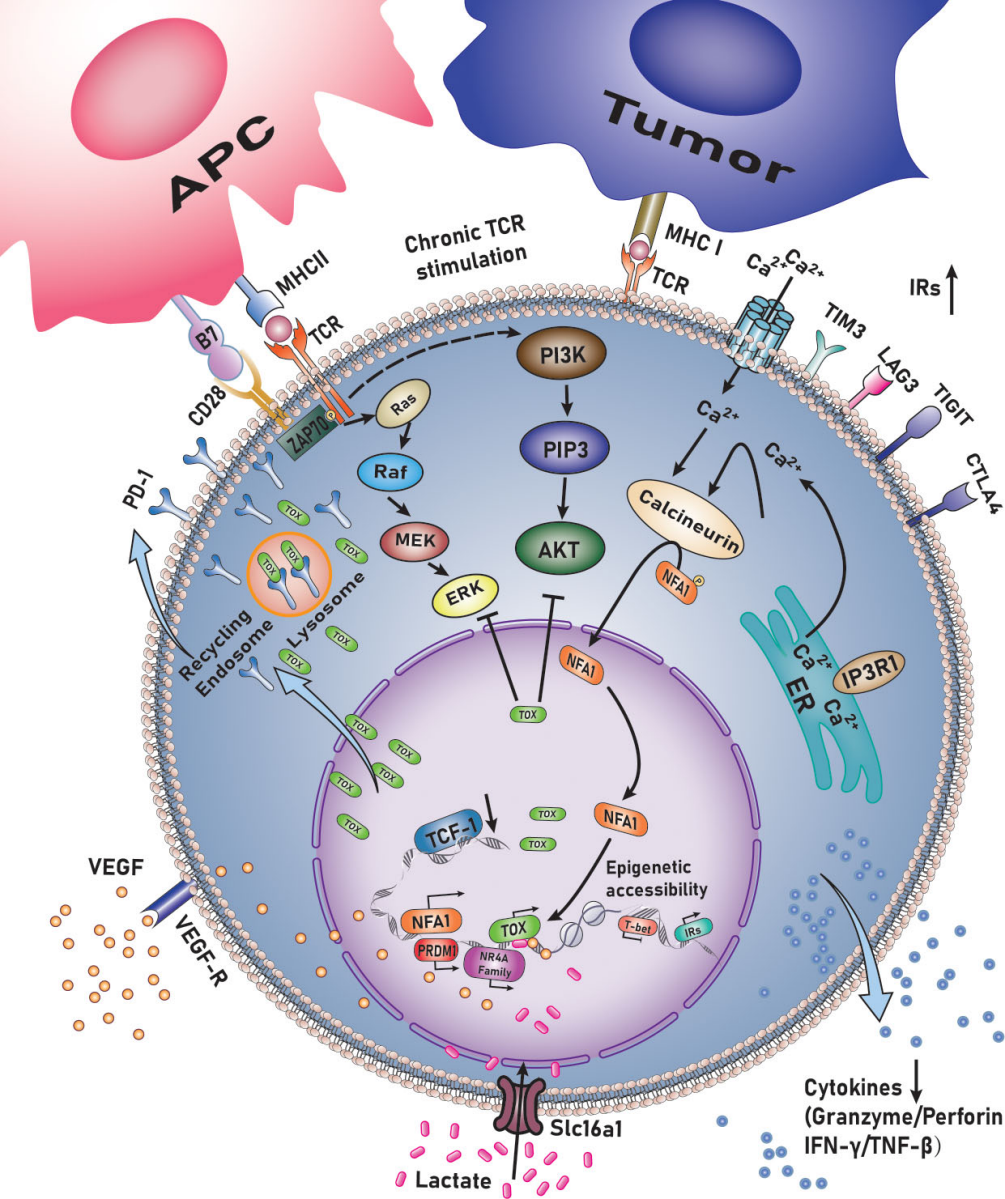
TOX regulates the exhaustion of CD8^+^ T lymphocytes.

TOX exhausts CD8^+^T lymphocytes in mouse models (infection (13–15) or tumor models) or human cancers, including melanoma, non-small cell lung carcinoma (16), colorectal cancer (17), liver cancer (18), bladder cancer, oral squamous cell carcinoma (19), acute myeloid leukemia (18, 20), multiple myeloma (21), and non-Hodgkin’s lymphoma (22) (**Figure 1**) (**Table 1**).

**Table 1:** TOX is intrinsically and extrinsically involved in malignancies.

Tumor type	Species	Tissue/Cell	Reference(s)
Breast cancer	H^1^	Tumor cells	(23–25)
	M^2^	TI^3^-CTLs	(23)
Lung cancer	H	Tumor cells	(24)
	M	TI-CTLs	(16, 23)
LUAD^4^	H	Tumor cells	(26)
CRC^5^	H	colon specimens	(27)
	H	Tumor cells	(28)
	H	TI-CTLs	(17)
	M	TI-CTLs	(13, 16, 29)
NSCLC^6^	H	TI-CTLs	(16)
HNSCC^7^	H	TI-CTLs	(16)
Liver cancer	H/M	TI-CTLs	(23)
	M	TI-CTLs	(29)
OSCC^8^	H	TI-CTLs	(19)
HCC^9^	M/H	TI-CTLs	(29)
melanoma	M/H	CTLs	(13, 14)
	M	TI-CTLs	(23, 29)
bladder cancer	H	CTLs	(30)
B/T-cell lymphomas	H	Tumor cells	(8)
B-NHL^10^	H	T cells	(22)
CTCL^11^	H	Tumor cells	(31–36)
CBCL^12^	H	Tumor cells	(37)
T-ALL^13^	Z^14^/H	Tumor cells	(38)
AML^15^	H	CD8^+^T	(18, 39)
	H	PBMNs^16^	(40)
MM^17^	H	T lymphocytes	(21)

^1^Human

^2^Mouse

^3^Tumor infiltrating

^4^lung adenocarcinoma

^5^colorectal cancer

^6^non-small cell lung cancer

^7^Head and neck squamous cell carcinoma

^8^oral squamous cell carcinoma

^9^hepatocellular carcinoma

^10^non-Hodgkin lymphoma

^11^Cutaneous T-cell lymphomas

^12^cutaneous B-cell lymphomas

^13^T-cell Acute Lymphoblastic Leukemia

^14^Zebrafish

^15^acute myeloid leukemia

^16^Peripheral Blood Mononuclear Cells

^17^Multiple myeloma

TOX supports CD8^+^ T-lymphocyte maladjustment by enhancing transcription and ensuring the survival of TCF-1^+^ progenitors. It induces the generation of a failed phenotype during chronic infection with lymphocytic choriomeningitis virus (LCMV)—post TCR activation—in mice and hepatitis C virus (HCV) in humans (15). During network analysis, TOX is considered a pivotal factor in differentiating memory CTLs with acute or chronic infections. Single-cell RNA sequencing (sc-RNA-seq) also revealed the significance of TOX in inducing T cell exhaustion. The expression of T cell-related genes *PD-1*, cytotoxic T-lymphocyte associated protein 4 *(CTLA4)*, T cell immunoreceptor with Ig and ITIM domains (*TIGIT*) and TNF receptor superfamily member 9 *(TNFRSF9)* was positively correlated with that of TOX—and by extension the degree of exhaustion—while the expression of naive-related genes was negatively associated with that of TOX. Transcription factor network analysis demonstrated that positive regulatory domain containing 1(*PRDM1*) was a critical TOX regulatory factor. The motif responsible for binding *PRDM1* in the TOX promoter was identified using JASPAR. Dual-luciferase reporter gene assay in Jurkat (human T lymphocyte cell line) demonstrated that *PRDM1* activates the transcrivation of *TOX* (16). Seo et al. observed consistent and marked upregulation of *TOX*, *TOX2*, and members of the *nuclear receptor 4A (NR4A)* family in all published comparisons of RNA-seq data originating from exhausted vs. control CD8^+^T cells. Exhaustion of CD8^+^T lymphocytes involves a significant positive feedback loop NFAT, TOX, and members of NR4A family transcription factors. Importantly, the expression of *TOX*, *TOX2* and members of the NR4A family was highly induced in CD8^+^CAR^+^PD-1^hi^1TIM3^+hi^ tumor-infiltrating lymphocytes (TILs) by the Cl/Cn-regulated nuclear factor of activated T cells (NFAT), even in the absence of activating protein-1, which is its partner protein (41). Exhausted CD8^+^T lymphocytes are heterogeneous in nature and can be divided into four stages based on the expression of *Ly108* and *CD69*, i.e., Tex^prog1^, Tex^prog2^, Tex^int^, and Tex^term^. The differentiation stage corresponds to TCF1^hi^Tox^hi^, TCF1^int^TOX^hi^, TCF1^hi^Tox^hi^, and TCF1^neg^T-box expressed in T lymphocytes (T-bet)^hi^Tox^int^, and finally to TCF1^neg^T-bet^lo^TOX^hi^Eomes^hi^. Thus, TOX may have a direct inhibitory effect on the expression of *T-bet* (13).

TOX also drives the epigenetic landscape of exhausted T lymphocytes (TEX) and establishes a exhaustion-specific epigenetic landscape. By comparing transcripts of virus-specific CD8^+^T lymphocytes with those of acute or chronic LCMV infection, it was found that the TOX-coding chromatin in TEX was more open and accessible during chronic viral infection, indicating epigenetic remodeling of TOX in TEX. The TOX coding site exhibits a dense open chromatin region harboring a “stretch”or “super” enhancer. Further analysis revealed that TOX expression is driven by continuous activation of chronic TCR, Cn, and *NFAT2*. However, forcing the *TOX* expression in *NFAT2*-cKO cells is sufficient for recovering the initial TEX differentiation, suggesting that TOX acts as a key *NFAT* downstream factor to differentiate TEX. Induction expression of *TOX* requires the expression of *NFAT2* and *TOX* at the beginning, while *TOX*-dependent TEX programs, once established, are no longer affected by Cn signaling (14). *TOX* may also regulate the epigenetic landscape by opening and increasing the accessibility, concurrently it could regulate the expression of transcription factors and their targets in TEX (indirectly). In fact, analysis of the interaction between transcripts and epigenetic markers indicated that Tox^-/-^T cells have fewer transcripts downstream of TCR signals (including *NFAT2*). Additionally, transcription factors (nuclear receptor subfamily 1 group D member 2 (*Nr1d2*), activating transcription factor 3 (*Atf3*), *Bcl6*, SRY-box transcription factor 4 (*Sox4*), etc.) could not be detected when *TOX* was knocked out. Thus, *TOX* synergizes with some transcription factors, which is seen as a paradigm to induce CD8^+^T lymphocytes exhaustion in the context of epigenetic landscapr and transcriptional remodeling. Deletion of *TOX* in tumor-specific T lymphocytes (TST) cancels the exhaustive procedure. When Tox is knocked out in TST cells, the expression of IRs (PD-1, ectonucleoside triphosphate diphosphohydrolase 1 (Entpd1), TIM3, 2B4, and Tigit)—the chromatin of most of which is inaccessible—is not upregulated, whereas *TCF-1* remains highly expressed. Loss of TOX in TST cells reverses the depletion process. It is noteworthy that once TOX is knocked out, CD8SP lymphocytes can differentiate into normal effector and memory cells under conditions of acute infection, while they do not persist in the tumor microenvironment (23).

Transcriptional analysis has identified a significant decrease in the viral titer in the blood and spleen of mice implanted with CTLs post LCMV infection. TOX-deficient TST cells lead to decreased expression of IRs and regain the ability to proliferate, while TOX-deficient TST cells still exhibit decreased cytokine production and cytotoxicity in the murine model of hepatocellular carcinoma.

Exhausted T lymphocytes demonstrate specific epigenetic and transcriptional features post chronic infection, and exhaustion-related epigenetic changes are not reversed after being cured. A study on post-cured HCV patients revealed that 77.1% of the scars persisted in a long-term follow-up, while only 18.2% decreased. It also revealed the presence of super-enhancers, including those near *TOX*, suggesting that the removal of chronic antigens and inflammation does not reshape specific epigenetic changes (42).

In addition to the above infection-related mouse models, TOX-mediated regulation of CD8^+^ lymphocytes dysfunction has also been verified in tumors or viral infections. Moreover, *TOX* and hypoxia inducible factor 1 subunit alpha (*HIF-1α*) are the key transcriptional regulators in the epigenetic depletion of T lymphocytes post curing of HCV-specific infections; these probably maintain exhaustion leading to the establishment of epigenetic scars. Consistent with this, after direct antiviral therapy, not only the surrounding scar but also the continued high expression of genes near ChARs is reversed. *TOX* regulates *T-bet* expression by maintaining a balance between the counts of Tex^int^ and Tex^term^ cells.

#### 2.4.3 All activated CD8^+^ T lymphocytes express TOX

Although further studies on *TOX*-driven CTL exhaustion have made breakthroughs, Sekine et al. (43) affirmed that *TOX* not only regulated T cell exhaustion, but was also detected in all activated CD8^+^ T lymphocytes, particularly in memory T cell subsets, including effect memory (TEM) T cells and terminal differentiation effect memory T cells, that re-express CD45RA (TEMRA). In Tox^+^TCF-7^+^cells, multiple effect memory genes are also expressed differently, including granzymes A and B, perforin 1*(Prf1)*, C-X3-C motif chemokine receptor 1*(Cx3cr1)*, *T-bet*, and eomesodermin *(Eomes)*. The TEM T cells with the highest TOX level were enriched with effect-related genes (C-C motif chemokine ligand 5 *(Ccl5)*, Gzma, natural killer cell granule protein 7 (*Nkg7*), and perforin 1(*Prf1*)). Another finding is that in dysfunctional TST cells, IR regulation is not universally coupled to dysfunction. Dysfunctional TCRTAG TILs with *TOX* knockout exhibit phenotypes and transcripts similar to those exhibited by effective *TOX*-TCROT1 TILs (23). Approximately 5-10% of the CD8^+^T lymphocytes express TOX (8). T cells expressing TOX in healthy donors also express functional molecules, including granzyme B, perforin, and cytokines, including IFN and TNF (43), suggesting that TOX could also be expressed by effective T lymphocytes, which is consistent with the results obtained in mouse studies (14, 15). Sekine et al. (43) also evaluated diverse types of CD8^+^ T lymphocytes against various viruses,i.e., influenza virus—which causes acute infection—cytomegalovirus (CMV), herpes virus (EBV), and human immunodeficiency virus (HIV), which cause chronic infection. Similar to the results obtained in mice infected with LCMV,TCF-1 expression is upregulated in acute infection, while TOX expression is upregulated after being infected with CMV, EBV, and HIV. Specific CD8^+^ effect memory T cells exhibiting higher TOX expression after infection with influenza virus, CMV, EBV, or HIV, suggesting that higher TOX expression is associated with more mature differentiation states of T cells. High expression of TOX, PD-1, and effective proteins (IFN-γ, granzyme B) in effective CD8^+^ T_mem_ cells indicate that cytokines drive T lymphocytes activity and TOX expression. Perhaps proinflammatory factors are competent enough to induce TOX expression and cause TOX heterogeneity. IL-12/15/18 and persistent TCR signal activation upregulate PD-1 and TOX expression in CD8^+^ T_mem_ cells. This difference is related to the heterogeneity produced by diverse stimulation signals (44). Researchers have attributed TOX heterogeneity to IL-15 expression in the microenvironment (35, 45). Conversely, vascular endothelial growth factor (VEGF)-A induces TOX expression to regulate T cell dysfunction, and TOX exhausts VEGF-A-treated T cells through functional impairment and *via* specific transcriptional processes (17). Furthermore, epigenetic evaluation revealed that future work determining the relationship between TOX and IRs will require the analysis of these inflammatory cues and other signals that can shape T_rm_ phenotypes, including co-stimulation and tetanic TCR signals. sc-RNA-seq techniques revealed the heterogeneity of CD8^+^T cells in mice. Two stem cell-like CD8^+^ memory T lymphocytes subsets, T_STEM_ and T_PEX_—CCR7^+^PD-1^-^TIGIT^-^ and CCR7^+^PD-1^+^TIGIT^+^, respectively—were identified. In-depth analysis demonstrated that T_PEX_ cells were in a state of functional inactivation compared with T_STEM_ cells—with respect to the expression of *TOX* and *TOX2*, expression as well—but their memory function was not lost, indicating their ability to adapt to continuous stimulation with antigens. Similarly, studies have found that TOX—as a key factor in autoimmune diseases—inhibits CD244 expression when effector T cells differentiate into SLECs, allowing them to cause autoimmune destruction. Another study on CD8^+^ T lymphocyte-mediated central nervous system inflammation demonstrated that TOX could impart CD8^+^T lymphocytes the ability to destroy tissues by hampering inhibitor of DNA binding 2 (*ID2*) activity. TOX attenuates the terminal differentiation of CTLs by modulating the *Id2*-, *TCF-1*-, and notch-driven pathways. In autoimmune models of chronic central nervous system inflammation, long-lived self-reactive T lymphocytes differentiate by fine-tuning their chromatin accessibility and securing a *TOX*-mediated gene expression program. In the CNS autoimmunity process, auto-reactive T lymphocytes adapt to chronic TCR stimulation while maintaining pathogenic activity and may affect other chronic immune-driven diseases. CTL-mediated oligodendrocyte death and resulting diseases is largely dependent on *TOX*. Page et al. demonstrated that that the expression of the DNA-binding factor, *TOX* promotes the brain development potential of pathogen-challenged CD8^+^T lymphocytes, and that its expression is determined by the microbial environment shaped by the CTLs (46).

## 3 TOX is intrinsically and extrinsically involved in the development of malignancies

### 3.1 Upregulated TOX in T cells is tumorigenic

The expression of TOX varies during all T lymphocytes differentiation stages. Mature CD4 or CD8 SP thymocytes tend to have low TOX expression. The necessity of TOX for CD4 lineage development suggests that its abnormal expression may lead to clonal proliferation of malignant T tumor cells. There is extensive evidence that abnormal TOX expression leads to lymphoma development (8, 22, 47), especially T-lymphoma. The effects of TOX on cutaneous T-cell lymphoma (CTCL) have been studied extensively (31–35). CTCL cells are CD4^+^CD8^-^, wherein TOX is overexpressed, but TOX expression is not tumor-specific as other phenotypes of CTCL cells, including CD4^+^CD8^+^ and CD4^-^CD8^-^, also express TOX. Higher TOX expression is related to worse prognosis (40) and can therefore be used as a potential therapeutic target (48)as well as a tool to assist in diagnosis (8). Abnormal TOX expression is found not only in T-ALL but also in solid tumors (colorectal cancer (CRC), breast cancer, and lung adenocarcinoma) (**Table 1**).

### 3.2 TOX regulates the tumor microenvironment

TOX regulates the functioning of several immune organs or immune cells. In this review, the critical role of TOX in regulating T lymphocytes subtypes and functioning is summarized. The occurrence and progression of tumors are a series of complicated processes—accompanied by changes in the tumor microenvironment—among which the immune response is an important component.

#### 3.2.1 TOX^+^CD4 subsets can enable the establishment of an immunosuppressive microenvironment

A small number of CD4SP thymocytes called regulatory cells (Tregs) (CD4^+^CD25^+^FOXP3^+^CD127^dim^) survive under conditions of strong negative selection (49). Higher counts of TOX^+^ Treg subsets in the bone marrow may be helpful in mediating immunosuppressive microenvironment in the bone marrow (7). The proportion of the TOX^+^ Treg subgroup is increased in patients with non-Hodgkin’s lymphoma (22), multiple myeloma (MM) (21), or *de novo* AML (18), and this phenomenon results in Treg activation instead of Treg exaustion. *Fusobacterium nucleatum* may aggravate rectal cancer by inhibiting antitumor T cell-mediated adaptive immunity. *Fusobacterium nucleatum* abundance in colorectal tumors is negatively correlated with CD4^+^T lymphocyte density and TOX expression. As colorectal cancer progresses toward metastasis, TOX expression decreases. TOX can also be utilized as a potential marker to distinguish between hyperplastic polyps (HPs) and sessile serrated adenomas (SSAs) (27).

In addition to being closely linked to the tumor microenvironment, TOX is also related to the tumor itself, which is the cause of tumor development and may therefore be a potential target of cancer therapies.

#### 3.2.2 TOX induced the counts of CTLs exhausted in tumors

CTL exhaustion leads to immune escape, which not only accelerates tumorigenesis but also serves as an important bottleneck for hematopoietic stem cell transplantation (HSCT) or CAR-T therapy. CTLs with high expression of TOX have been reported in diverse tumor models in humans and mice (**Table 1**). The role of TOX in CD8^+^ lymphocyte heterogeneity was revealed using advanced techniques, such as sc-RNA-seq or an assay for transposase-accessible chromatin with Assay for Transposase-Accessible Chromatin using sequencing(ATAC-Seq).

In patients with melanoma and non-small cell lung cancer, TOX expression is significantly higher in CD8^+^T lymphocytes, a phenomenon that is positively correlated with the expression of PD-1, TIM3, CTLA4, and other IRs. As noted above, ineffective T lymphocytes are enriched in IRs modulated by TOX. Importantly, TOX expressed by CTLs is negatively correlated with overall survival and response to anti-PD-1 therapy (16). Variations in TOX expression in CTLs in bladder cancer patients result in radically different responses toward anti-PD-1 and TIGIT inhibitors (30). Immune checkpoint inhibitors restore the effect of exhausted CD8^+^T lymphocytes *in vitro*. PD-1^high^CD8^+^ T lymphocytes maintain the highest expression of iIRs on their surface, with characteristics of terminal failure and the highest tumor antigen reactivity. TIGIT^+^CD73^-^CD39^+^CD8^+^ T lymphocytes expressed TOX in high levels in newly diagnosed AML patients, which was further increased in patients with recurrent AML. The higher the percentage of PD- 1^high^TOX^+^CTLs in patients with bladder cancer, the more exhausted the phenotype of the lymphocytes (39). Similar findings have been reported in patients with MM (21). GO analysis demonstrated significant enrichment in the *PD-1* signal-related genome after the induction of TOX overexpression, whereas *TOX* gene inhibition was significantly enriched in the PI3K pathway. Furthermore, KEGG analysis revealed that *TOX* overexpression in TIL-CD8^+^ lymphocytes could suppress the Ras and PI3K-AKT pathways. TOX may bind to PD-1 in the cytoplasm, thereby preventing PD1 from becoming the target of lysosome-mediated degradation and leading to high PD-1 expression on the surface of CTLs (29). TOX may also be used as a strong predictor to stratify at-risk patients and predict their therapeutic response to anti-PD-1 mAbs or combination anti-TIGIT mAbs.

As a key upstream regulatory factor affecting CTL exhaustion, TOX may be a potential target for tumor therapy. Moreover, it can also prolong survival and predict the response to immunosuppressants, including PD-1 or PD-1 inhibitors combined with TIGIT blockers. PD-1 inhibitors combined with TIGIT blockers can restore the activity of exhausted CD8^+^ PD-1^hi^TOX^+^ T lymphocytes. CAR-T cell therapy is also a potential landmark therapy, but is also affected by the autologous landscape. However, *TOX* and *TOX2*-deleted CAR-expressing tumor-infiltrating T lymphocytes (TILs) are superior to *TOX*- or *TOX2*-deleted WT CAR TILs or CAR TILs in terms of clearing human CD19^+^ tumor cells (41). To resolve the dilemma of no small molecule inhibitors targeting TOX, Agrawal et al. selected 140 compounds from 7.6 million compounds against *TOX via* computer aided drug design (CADD) (48). Targeting antigens to red blood cells *in vivo* can result in the effective presentation of antigens to T cells, and these T cells demonstrate upregulation of TOX and PD-1, which was associated with the exhausted phenotype by transcriptional analysis. Maestre et al. (8) prepared a new anti-*TOX* monoclonal antibody (mAb) (clone NAN448B) utilizing the HIS tag produced by the ES21 strain as an antigen and immunizing Wistar rats with *TOX* fusion proteins containing 250 amino acid residues. A single low dose of red blood cell-related antigen is sufficient to initiate depletion-related procedures (50), which has contributed to the development of new interventions for autoimmune diseases.

## 4 Conclusion

T lymphocytes gradually differentiate from HSCs into mature T lymphocytes, which undergo a series of complex and precise regulatory processes. During this process, is a minor change in the expression of TOX and it plays a pivotal role in thymic selection, positive selection, negative selection, central tolerance, and T lymphocytes lineage commitment.

TOX expression demonstrated heterogeneity in diverse species, and with respect to various stages of cell development, stimulation signals (cytokines/VEGF-A/TCR), and pathogens (influenza virus/LMCV/HCV/HIV/EBV/CMV). Further understanding of the mechanisms by which TOX regulates T lymphocytes differentiation into various subsets and its dependent protein network could yield new insights for use in immunotherapy, which is a key step toward the development of safer and more effective T-cell therapies or combination therapies for solid cancer.

## Author contributions

HN wrote and revised the manuscript. HW conceived this review and is accountable for all aspects of the work. All authors contributed to the article and approved the submitted version.
